# Letter from Philadelphia

**Published:** 1853-09

**Authors:** J. C. Hughes

**Affiliations:** Philadelphia


The following letter of Dr. Hughes should have had precedence in the order of time, to the one published in our last; but as we were desirous of laying before our readers a synopsis of the proceedings of the American Medical Association, it was thought proper to give it a prior insertion.

LETTER FROM DR. HUGHES.

Philadelphia, May 1st, 1853.

Gentlemen:

I have been absent from you as well as from my Western home, for nearly three months; and during this time have met and mingled with many of my professional brethren, while on duty as well as in the social circle. I have also visited nearly all the Medical Institutions of the East, as well as the different Hospitals of these Cities, noticed the mode and manner of conducting them, and the advantages to be derived therefrom.

I shall endeavor therefore to give you as short and succinct an account of my trip, the profession, men and things, as this sheet will permit.

I have nothing of interest professionally, until I reach Baltimore; and allow me here to say, that past scenes and associations came up to my memory with a thousand endearing recollections, when after an absence of eight years, I again reached a city I so much loved, and where I had spent so many pleasant as well as profitable days in the study of my profession. How pleasant once more to meet my preceptor, and take by the hand those who had so kindly led me on, step by step, through the labyrinths and mysteries of the science, stripping from it the veil which obscured it from my view, that I might understand, and again to enter the time-honored halls of my *alma mater*, where joy to many a heart has flown, as well as *green room* grief.

This institution is still in a flourishing condition. The Faculty is composed of good men, who are distinguished in the medical world, and whose names are connected with the Medical literature of our country. The names of Smith, Chew, Roby, Aiken and Miltenberger, are familiar to us, as old teachers and prominent men, and those who have been lately added to the number, are no less conspicuous. The attention I received while there, and the interest manifested in behalf of our Institution, was gratifying in the extreme.

I feel under deep obligations to Prof. Nathan R. Smith, for the aid extended me in procuring many of the models which I have procured. Most of those representing important dislocations as well as deformities, were obtained from moulds of those forming his extensive collection. Those representations of all the dislocations, fractures and deformities, will add much to the interest of my department during the coming winter.

The Hospital in connection with the Institution and under the control of the Faculty, has been much enlarged, and is under the immediate direction, at this time, of Prof. Miltenberger, with whom I frequently visited it, and who in connection with the resident physician, extended to me every means of improvement the institution afforded.

The Washington University is still in existence, but the number of students in attendance during the last session was small.

Among the younger members of the profession in the city, who occupy prominent positions, we might mention the names of Dunbar, Bond, Smith, Perkins and Donalson, the latter two having during the last year spent a great portion of their time in Europe. Dr. Smith has the position of Demonstrator of Anatomy in the University, and bids fair to occupy, at some future day, as proud a position as his father. Dr. Dunbar has devoted much time to teaching, and is at this time engaged in delivering a summer course to a very respectable class. His Cabinet is very extensive, and among his collections on

Anatomy, I had the pleasure of seeing a number of my own preparations.

The Alms House of the city and county, situated some two miles from the city, is in a prosperous condition. It is under the direction of Governors appointed by the city and county. There are now within its walls some six hundred paupers, who are medically under charge of Dr. Donalson and his colleague, assisted by the house physicians, six in number, worthy and competent young men. This Institution I visited frequently by invitation of the attending physicians, and by the assistance of the resident physicians, took some casts of very rare and interesting surgical cases.

Having closed my visit in the Monumental City, I took the cars, and after a ride of four hours reached this city of brotherly love.

Philadelphia claims, and I might say justly too, to be the fountain of Medical Science, from which flow the purest as well as deepest medical truths. Scarcely had I found a resting place, when I was attacked by disease, which proved of a bilious character, keeping me confined to my room for several days. I was indeed very ill, but having called to my aid Prof. J. K. Mitchell, and having the kind attention of my hostess, as well as of numerous friends, I soon began to convalesce.

As soon as health would permit, I called upon a number of the profession, to whom I had letters of introduction. Having received a letter from Mr. Hiatt of Keokuk, to his friend Dr. Hunt, who for the last two years has been one of the house physicians to the Pennsylvania Hospital, I was immediately introduced to Drs. Neil, Pepper, Norris, Fox, and others, constituting the medical staff of that benevolent institution, and had extended to me a free ticket to its wards and library during my stay in the city. This Institution is among the oldest of our country, was established by Penn himself, and is still under the direction of his followers. The building is admirably arranged in every respect, and its government is most perfect. Its location is central and in a beautiful portion of the city, occupying an entire square. The patients in the house at this time, number about 250 and are principally recent cases, and the greater number surgical. I visit it daily, and have had the opportunity of seeing every variety of disease both medical and surgical. The apparatus used in cases of dislocations and fractures, is such as I have heretofore used, with some slight modifications. I have ordered from the manufacturer, in connection with the institution, a full set, which I shall have to present to the class the coming winter. While in Baltimore I also procured Prof. Smith's apparatus, which in many cases is preferable to any other. Two of the physicians, composing the medical staff, visit the institution daily. Drs. Neil and Pepper are now on duty, the former as Surgeon and the latter as Physician. Dr. N. is a young but successful operator; he has been connected with the University as Demonstrator, and was a candidate for the Chair of Anatomy, made vacant by the death of Prof. Horner, but was defeated by Dr. Leidy. Dr. P. is familiarly known to the profession as a most thorough medical scholar. The house physicians and apothecary are young men of high medical attainments.

The Medical Institutions of the city are numerous. The Jefferson School and University stand most prominent, and in point of numbers, surpass any other schools of the country. The Faculties of these institutions, are well known to the profession as being composed of prominent and scientific men. The Pennsylvania Medical College stands third in point of reputation and numbers, and has an able Faculty; among the number you will recognize the names of Smith and Allen, both substantial men. Fourth we might class the Philadelphia College, a part of whose Faculty are familiarly known to the profession. McClintock stands at the head and I might say owns and governs it. Other schools have been chartered, but as yet are in the back ground. Of the Female College and its Professors, I can say but little, as I have not had the pleasure of an acquaintance with any one in connection with it. There seems to be more known and said abroad concerning it, than at home.

Several spring and summer schools have been established, conducted principally by the younger members of the profession, many of whom are prominent, and whose names will at some future day be connected with the older and more prominent institutions. Apart from all her schools, Philadelphia has a host of medical talent to boast and be proud of.

The object of my visit being more in connection with Surgery, and particularly operative Surgery, and finding this branch of the practice, and especially the capital operations in the hands of Professors Mutter and Pancoast, the former Professor of Surgery and the latter of Anatomy in the Jefferson school, and having had invitations from them to witness their operations in private practice, as well as before the class, I availed myself of their courtesies, and I must say that the kindness and attention bestowed upon me by them as well as by other members of the Faculty is most flattering.

I am witnessing operations, as well as other surgical cases of interest daily. Had I time and space I would report some of them, and may do so in the future.

The Blockley Hospital and Alms House, of the City and County of Philadelphia is very extensive. It is situated some distance from the city, upon the eastern bank of the Schuylkill. The building is three stories high, forming a hollow square, and of sufficient size to accommodate from two to three thousand patients. A part is devoted exclusively to the reception of insane cases, another part as a lying-in Hospital and Nursery. A third part to Syphilitic cases, and the balance for the poor people. The building has been erected for a number of years, and has not the advantages of recent improvements in the manner of heating and ventilation, which are necessary in such buildings, for the comfort and health of the unfortunate class of beings who are forced to occupy it. The medical arrangement differs somewhat from the Maryland Institutions. It is under the direction of one physician, who resides in the house and is paid a salary of $3,000 per annum, assisted by eight young physicians who are also residents of the house, but receive no remuneration for services.

In connection with the profession of the city, I must not close before mentioning the course of instruction by Dr. Warrington. Dr. W. has been for many years devoted to the profession. He has had charge of the lying-in Hospital of the city for many years, and is at this time delivering a course of lectures on that subject to a class composed of males and females. His instructions are principally of a practical character. The females in attendance take the part of nurses, and are instructed in that department alone.

I might say much more in connection with institutions and profession of the city, but must close for the present.

Yours, truly, J. C. HUGHES.

